# EGCG-Mediated Potential Inhibition of Biofilm Development and Quorum Sensing in *Pseudomonas aeruginosa*

**DOI:** 10.3390/ijms22094946

**Published:** 2021-05-06

**Authors:** Suqi Hao, Dan Yang, Ling Zhao, Fei Shi, Gang Ye, Hualin Fu, Juchun Lin, Hongrui Guo, Ran He, Jianlong Li, Hongwei Chen, Muhammad Faraz Khan, Yinglun Li, Huaqiao Tang

**Affiliations:** 1 College of Veterinary Medicine, Sichuan Agricultural University, No. 211 Huimin Road, Wenjiang District, Chengdu 611130, China; HHQ100608@163.com (S.H.); ydan468317374@163.com (D.Y.); lingzhao@sicau.edu.cn (L.Z.); fei_shi@sicau.edu.cn (F.S.); yegang010101@163.com (G.Y.); fuhl.sicau@163.com (H.F.); juchunlin@126.com (J.L.); guohongrui@sicau.edu.cn (H.G.); ranhe1991@hotmail.com (R.H.); 2 College of Food Science, Sichuan Agricultural University, Chengdu 611130, China; jlli999@foxmail.com; 3 College of Veterinary Medicine, Southwest University, Chongqing 402460, China; dyxchw@swu.edu.cn; 4 Department of Botany, Faculty of Basic and Applied Sciences, University of Poonch Rawalakot, Rawalakot 12350, Pakistan; mfarazkhan@upr.edu.pk

**Keywords:** anti-quorum sensing, EGCG, *Pseudomonas aeruginosa*, virulence factors

## Abstract

*Pseudomonas aeruginosa* (*P. aeruginosa*), one of the dangerous multidrug resistance pathogens, orchestrates virulence factors production through quorum sensing (QS). Since the exploration of QS inhibitors, targeting virulence to circumvent bacterial pathogenesis without causing significant growth inhibition is a promising approach to treat *P. aeruginosa* infections. The present study has evaluated the anti-QS and anti-infective activity of epigallocatechin-3-gallate (EGCG), a bioactive ingredient of the traditional green tea, against *P. aeruginosa*. EGCG showed significant inhibitory effects on the development of biofilm, protease, elastase activity, swimming, and swarming motility, which was positively related to the production of C4-AHL. The expression of QS-related and QS-regulated virulence factors genes was also evaluated. Quantitative PCR analysis showed that EGCG significantly reduced the expression of *las*, *rhl*, and *PQS* genes and was highly correlated with the alterations of C4-AHL production. In-vivo experiments demonstrated that EGCG treatment reduced *P. aeruginosa* pathogenicity in *Caenorhabditis elegans* (*C. elegans*). EGCG increased the survival of *C. elegans* by 23.25%, 30.04%, and 36.35% in a dose-dependent manner. The findings of this study strongly suggest that EGCG could be a potential candidate for QS inhibition as an anti-virulence compound against bacterial infection.

## 1. Introduction

Tea is one of the most favorite beverages owing to its diversified benefits for humans [1]. The major health-enhancing ingredients of green tea are polyphenolic catechins, namely, epigallocatechin-3-gallate (EGCG), epigallocatechin (EGC), epicatechin-gallate (ECG), and epicatechin (EC). Among these, EGCG is the most abundant with the maximal share of total catechin contents [2]. EGCG contains a typical flavone-3-olpolyphenolic compound with eight hydroxyl groups that facilitates its diverse bioactive potential as an antioxidant, antiviral, anti-inflammatory, and anticancer agent [3,4,5,6]. Traditional Chinese green tea and its constituents have long been studied for antimicrobial properties. EGCG, for example, is reported to have growth inhibitory effects both against gram-positive and gram-negative bacteria, i.e., *Staphylococcus aureus* [7,8,9], *Escherichia coli* [10], *Acinetobacter baumannii* [11], and *klebsiella pneumoniae* [12].

QS is a cell-to-cell communication system that enables bacterial populations to modify behaviors according to the population density. It is moderated by the production of extracellular chemical signaling molecules, called autoinducers (AIs). The concentration of AIs will increase with the increase in bacterial population density, which, in turn, activates the homologous protein and regulates relevant gene expression. AIs also regulate some of the fundamental bacterial functions, including bioluminescence, biofilm formation, and virulence factor production [13,14]. There are three types of AIs in bacteria. Gram-negative species produce N-acyl L-homoserine lactone (AHL) whereas, Gram-positive species use small peptide signals and AI-2 for inter-species communication [15]. A body of evidence suggests that QS is essential for bacteria to control the production of virulence factors.

The rise of antimicrobial resistance (AMR) is a concerning threat to human health globally. AMR is defined as the ability of bacteria to grow in the presence of a drug that is supposed to kill them. Normally, antimicrobial compounds directly kill pathogens or suppress bacterial growth, which imposes a strong selective pressure. This is the main reason for the rapid growth of resistance in pathogenic microorganisms as they tend to evolve and evade the life threat, thereby acquiring resistance [16,17]. Thus, additional approaches are urgently needed to confront the resistant pathogens. Virulence factors are crucial for infection; therefore, interference with the production of virulence factors seems an advantageous therapeutic strategy [18,19]. Thus, QS inhibition is a promising area to explore anti-virulence drugs designed to intercept the expression of target genes [20].

*P. aeruginosa* is an opportunistic pathogen causing life-threatening infections, especially in patients suffering from cystic fibrosis, burn wounds, and those who are immunocompromised [21,22]. Chronic *P. aeruginosa* infections are usually treated with tobramycin, meropenem, or ciprofloxacin [23]. *S. aureus* is another noxious pathogen commonly associated with secondary infections in cystic fibrosis patients. Mixed infection of *P. aeruginosa* and *S. aureus* increases patient morbidity [24]. Various anti-staphylococcal strategies such as antimicrobial peptides (AMPs), enzybiotics, antivirulence agents, gene editing enzymes, bacteriophages, and nanoparticles (NPs) are being developed as alternative therapeutic options to antibiotics [25]. Modifying the chemical structure of an antimicrobial agent and synthesis, new compounds for inhibiting resistant mechanisms to antibiotics is also a novel method to exploit new antibacterial agents [26]. Aamer Saeed et al. reported H-BDF, a guanidine derivative bearing adamantane-1-carbonyl and two 2-bromo-4,6-di-fluoro-phenyl groups, was able to significantly potentiate antibacterial synergy with meropenem and ciprofloxacin [27]. Both *P. aeruginosa* and *S. aureus* are included in the list of multidrug-resistant (MDR) bacteria referred to as the “ESKAPE” (*Enterococcus* spp., *S. aureus*, *Klebsiella* spp., *Acinetobacter baumannii*, *P. aeruginosa*, and *Enterobacter* spp.) pathogens [28]. Drug resistance is primarily attributed to some efflux pumps adapted specifically to eliminate drugs from the cells, making it hard to eradicate pathogenic infections [29,30]. In addition, biofilm is also an adaptation strategy in antibiotic resistance that acts as a protective barrier against antibiotic penetration [31]. Antifungal ketoconazole has enhanced the activity of fluoroquinolones against MDR *S*. *aureus* via inhibiting efflux pump and biofilm formation in vitro. The cephalosporin nitric oxide (NO)-donor prodrug DEA-C3D (‘DiEthylAmin-Cephalosporin-3′-Diazeniumdiolate’) was also reported to be able to disrupt biofilms formed by three *P*. *aeruginosa* clinical isolates. 

DEA-C3D in combination with colistin was also found to prevent biofilm regrowth [32]. Despite some clinical gains, the treatment of *P. aeruginosa* infection is still a universal problem.

*P. aeruginosa* has become one of the classical model organisms in QS research. *P. aeruginosa* is composed of four interconnected systems (LasI/LasR, RhlI/RhlR, PQS, and IQS). Each system utilizes a particular signal, including N-(3-oxododecanoyl)-L-homoserine lactone (3-oxo-C12-HSL), N-butanoyl-L-homoserine lactone (C4-HSL), quinolone-based signaling system (PQS), and the quorum-sensing signal (IQS). The four QS systems of *P. aeruginosa* have distinct hierarchical structures, with the LasI/LasR system being primary. QS regulates a number of virulence factors in *P. aeruginosa*, including pyocyanin, rhamnolipids, LasB elastase, swarming motility, and biofilm formation [33,34]. Therefore, QS inhibitors are a highly effective therapeutic strategy by suppressing the expression of virulence factors to attenuate pathogens infection.

The emphasis of previous studies primarily aimed to evaluate the antibacterial activity of EGCG and potential mechanism of possible synergism with antibiotics. Little research was conducted based on the QS inhibition activity of EGCG to explore the ability to attenuate bacterial infection. The objectives of this study were as follows: Firstly, we determined the effect of EGCG on the QS-related phenotypes in vitro, including biofilm formation and virulence factors. Secondly, we studied the mechanism of action of EGCG on the *P. aeruginosa* QS system at the gene level. Lastly, the QS-based anti-infective activity was tested in vivo using a *C. elegans* model. Our paper can provide more clues to the benefits of tea drinking based on the QS inhibition activity of EGCG.

## 2. Materials and Methods

### 2.1. Bacterial Strains, Growth Conditions and Chemicals

*P. aeruginosa* (PAO1), and *Chromobacterium violaceum (C. violaceum*) ATCC 12,472 and ATCC CV026 were cultured in Luria Bertani (LB) medium (Sangon Biotech, Shanghai, China). Before experiments, both *P. aeruginosa* and *C. violaceum* strains were streaked from −80 °C frozen culture stocks. *P. aeruginosa* single colonies were incubated in LB broth and cultured 16 h in LB broth at 37 °C at 170 rpm. *C. violaceum* was cultured in LB broth at 30 °C at 160 rpm. EGCG was obtained from Shanghai Yuanye Bio-Technology Co., Ltd. (Shanghai, China) and dissolved in dimethyl sulfoxide (DMSO) (Biosharp).

### 2.2. Bioassay for Anti-Quorum Sensing on Solid Broth

Quorum sensing inhibition (QSI) assay was performed with a few modifications in the procedure described previously [35]. An overnight culture of *C. violaceum* 12,472 was diluted to 10 mL of molten agar in a ratio of 1:100; after becoming solidified, different concentrations of EGCG were added to the wells (6 mm in diameter). Next, the plates were incubated at 30 °C for 24 h. QSI was assessed from the formation of a ring of colorless but viable cells around the well.

### 2.3. Bioassay for Anti-QS Activity in Liquid Broth

A violacein production quantitative assay was used for measuring anti-QS activity. Briefly, overnight grown cultures were inoculated into LB broth supplemented with EGCG in 96-well plates. After incubation at 30 °C for 24 h. The cells were lysed by adding 150 μL of 10% sodium dodecyl sulfate (SDS), followed by mixing for 10 s with a vortex mixer. Violacein was extracted from the cell lysate by adding 600 μL of water-saturated butanol, vortexing for 5 s, and centrifuged for 5 min at 12,000 rpm. The butanol phase (upper) containing the violacein was transferred into 96-well plates, with 150 μL of extract per well. The plates were analyzed at 585 nm using a microtiter plate reader. The effect of EGCG was determined based on the relative levels of violacein production, and the control value was set to 100% [36].

### 2.4. Determination of Minimum Inhibitory Concentration (MIC) and Growth Curve

MIC of the EGCG (≥98%) against *P. aeruginosa* was determined by using the broth microdilution method, following the guidelines of clinical and laboratory standards institute (CLSI), USA, 2006. Briefly, overnight grown cultures (5 × 10^5^ CFU) of the test organisms were dispensed into LB broth supplemented with the test compounds with concentrations ranging from 512 to 8 μg/mL. The MIC was calculated as the lowest concentration of the EGCG that completely inhibited visible growth. The sub-MIC of the EGCG was employed in all the assays of the present study [37].

### 2.5. Bacterial Growth Curve Assay

Growth curves were assessed according to the method described by Husain [38]. Overnight culture of *P. aeruginosa* was diluted in fresh LB medium (50 mL) to achieve a CFU of 5 × 10^5^. Different concentrations of EGCG, 256, 128, 64, and 0 μg/mL, were incubated with *P*. *aeruginosa* culture at 37 °C with continuous shaking (170 rpm). The culture suspension devoid of the test compounds was maintained as a control. Subsequently, 1 mL of the broth culture was withdrawn at every 2 h interval, and the turbidity was recorded at 600 nm to generate a growth curve.

### 2.6. Biofilm Inhibition Assay

The quantification of biofilm formation was assessed using regular 96-well microtiter plates. Inhibition of biofilm formation was studied as described by Chatterjee, with some modifications [39]. Briefly, *P*. *aeruginosa* was cultured in 96-well plates with different concentrations of EGCG (0, 64, 128, and 256 μg/mL) for 24 h, 36 h, and 48 h at 37 °C. The cultures were removed, and the plates were washed three times with PBS. The resulting biofilms were stained with 0.1% crystal violet for 15 min; unbound stain was rinsed twice with water. The plates were dried, and biofilms bound to crystal violet were solubilized with 200 μL of 95% alcohol. The absorbance was measured at 595 nm.

### 2.7. Quantitation of Extracellular Virulence Factors

For cell-free quantification of virulence factors, pyocyanin was extracted from the culture supernatant with chloroform (800 μL) at a ratio of 3:2 and re-extracted with 200 μL of 0.2 M HCL. Absorbance was recorded at 520 nm, as the production of pyocyanin multiplies the OD value by 17.072 [40]. Protease activity was determined with a 2% azocasein solution prepared in 1 M Tris-HCl buffer at pH 7.8. The substrate and culture supernatant was incubated at 4 °C in 5:3 ratio for 4 h in a reaction volume of 400 µL. Next, 500 μL of 10% trichloroacetic acid was added to stop the reaction, and the cultures were spun at 10,000× *g* for 10 min. Finally, 500 μL of NaOH was mixed with the suspension, and the absorbance was measured at 440 nm [41]. We also conducted the qualitative analysis of elastase activity as follows Skim milk agar plates containing 10 mL of 15% (*w/v*) skim milk, and 90 mL of LB nutrient agar were sterilized at 115 °C for 30 min. Holes were made on the milk agar plate. Culture supernatants (50 uL) of *P. aeruginosa* were added in a hole and incubated at 28 °C for 24 h. Proteolytic activity was calculated as a measure of the diameter of the clear zone surrounding the holes.

Rhamnolipids were extracted from culture supernatant with ethyl acetate at a 1:1 ratio, vortexed for 15 s, and centrifuged (10,000× *g*, 4 °C, 5 min). The upper layer was removed, and ethyl acetate extraction was repeated three times for each sample. The combined upper layer was left to evaporate overnight, and then 900 μL of orcinol reagent (0.19% orcinol in 53% H_2_SO_4_) was added to the precipitate, which was incubated at 80 °C for 30 min, absorbance was recorded at 420 nm and calculated as the content of rhamnolipids and the proportion between the rhamnose and rhamnolipid [42].

### 2.8. Swimming and Swarming Motility Assays

Swimming and swarming motility assays were conducted as previously described [43,44] with some modifications. Briefly, 2 μL of *P. aeruginosa* overnight culture containing EGCG at 0, 64, 128, 256 μg/mL were inoculated at the center of swimming (nutrient broth 1%, glucose 0.5%, and agar 0.3%) and swarming (0.3% agar, 1% tryptone, 0.5% Yeast extract powder, and 0.5% sodium chloride) plates. The plates were incubated at 37 °C for 24 h, and the diameter of the motility zones was measured.

### 2.9. Detection of the C4-AHL Molecular Production

Detection of the C4-AHL production was carried out according to the modified method previously reported [45]. Briefly, we mixed the culture supernatant of *P. aeruginosa* with an equal volume of ethyl acetate, vortexed for 15 s, then transferred the top supernatant to a new tube and repeated three times. The combined ethyl acetate fractions were evaporated, and the precipitate was re-suspended in 200 μL of DMSO. Samples were used immediately or freeze-dried and stored at −20 °C until required.

AHL extracts were tested by using the *C. violaceum* CV026 strain in a well diffusion assay. The overnight culture of *C. violaceum* CV026 was incorporated into semi-solid LB medium plates (10 g peptone, 10 g NaCl, 5 g yeast extract, 1000 mL distilled water, and 5 g agar) by dilution (1:100). Four evenly distributed wells (6 mm diameter) were made in the semi-solid agar plates. Test AHL extracts (or controls) were added to the well. The plates were then incubated at 30 °C for 24 h. Distances of violacein inhibition, as determined by the extent of purple coloration, were then measured in millimeters.

### 2.10. Gene Expression of QS Signal Molecular and Virulence Factors by Quantitative RT-PCR

Total RNA was extracted from the cultures using TRIzol (TRIzol reagent, Songon Biotech, Shanghai, China), and cDNA synthesis was conducted using a kit (ThermoFisher Scientific, Waltham, MA, USA, K1622) according to the manufacturer’s instructions. The concentration and purity of RNA were determined using the UV spectrophotometer (Implen, Munich, Germany). qRT-PCR was performed using the LightCycler^®^ 480II Master (Roche, Germany) with a final reaction volume of 10 μL. The expression of target genes was normalized to the expression of pvdq, which was used as a housekeeping gene. Primer sequences are listed in Table 1.

### 2.11. Virulence of P. aeruginosa Supernatant Treated by EGCG on C. elegans

The wild-type *C. elegans* strain (N2) and *Escherichia coli* (OP50) strain were obtained from the Caenorhabditis Genetics Center (CGC). Worms were synchronized by hypochlorite treatment of gravid adults. Synchronized worms were grown to L4 or young adult stage by incubating them at 20 °C in Nematode Growth Medium (NGM) for supernatant Virulence assays. After pouring of NGM plates, the overnight culture of cell-free supernatant was incubated at 37 °C for 12 h to form a lawn of bacteria with *E. coli* at a 1:1 ratio blended in a reaction volume of 200 µL. After incubation, the supernatant was allowed to reach room temperature before seeding with 30 young adult worms in triplicates. Percentage survival of the infected worm population was determined every 24 h interval during incubation at 20 °C.

### 2.12. Test the Anti-Infection Activity of EGCG on C. elegans

Briefly, Corresponding concentrations of EGCG were added to the NGM and were poured onto the plates. After plates were solidified, 200 µL of an overnight culture of *E. coli* or *P. aeruginosa* were seeded and incubated at 37 °C for 24 h to form lawn of bacteria, then allowed to reach room temperature before seeding with 30 young adult worms and conducted in triplicates. Percentage survival of the infected worm population was determined every 24 h interval during incubation at 20 °C [46].

### 2.13. Statistical Analysis

All experiments were carried out in triplicate, or sextuplicate, and values were expressed as means ± standard deviations (SD). The culture without EGCG treatment served as a vehicle control then compared with the EGCG treatment group for all statistical analyses by one-way ANOVA and *t*-tests using the SPSS. Differences with a *p* < 0.05 were considered statistically significant.

## 3. Results

### 3.1. QSI Assays of EGCG

**Figure 1:**
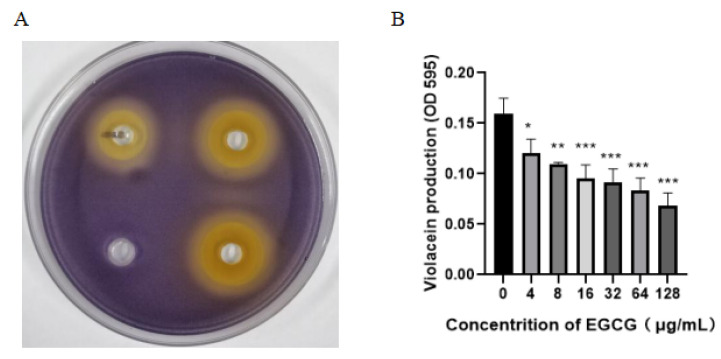
QSI effect of EGCG on biosensor *C. violaceum* 12472. (**A**) Visual qualitative assay for inhibition of *C. violaceum* violacein. (**B**) Quantitative estimation of violacein. (All data are representative of three independent experiments performed in triplicate and expressed as the mean  ±  SD values in each bar. * *p*  <  0.05; * *p  <  0.01; *** *p*  <  0.001).

As shown in Figure 1A, for qualitative assay, EGCG efficiently inhibited the violacein production. The size of zones was 23, 25, and 27 mm, respectively. On the contrary, the diameter of control was zero, which visually reflected EGCG was a valid QS inhibitor. EGCG also obviously degraded the violacein production in the quantitative experiment below the sub-mic (Figure 1B).

### 3.2. EGCG Inhibits P. aeruginosa Biofilm Formation

**Figure 2:**
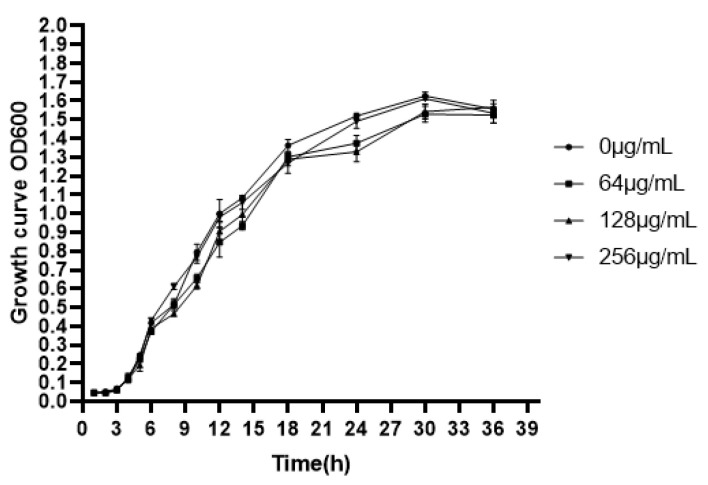
Effect of EGCG on the growth of *P. aeruginosa*. (All data are representative of three independent experiments performed in triplicate and expressed as the mean ± SD values in each bar.

**Figure 3:**
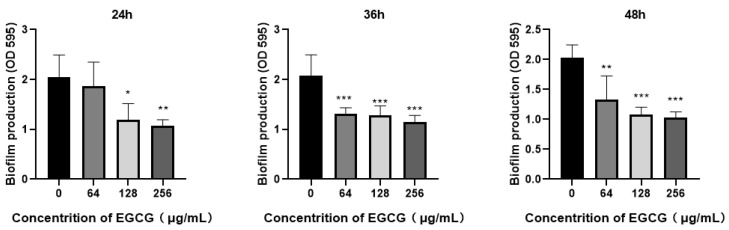
Concentration-dependent block effects of EGCG on *P. aeruginosa* biofilm formation. (All data are representative of three independent experiments performed in triplicate and expressed as the mean  ±  SD values in each bar. * *p*  <  0.05; ** *p*  <  0.01; *** *p*  <  0.001).

Chronic infectious effect of *P. aeruginosa* is mainly by reason of the formation of biofilm with attachment on a surface that is either biotic or abiotic [47,48]. Biofilm runs through the chronic infections of *P. aeruginosa*, and virulence factors play an important role in the process of biofilm development [49]. Our results indicated that the MIC of EGCG against *P. aeruginosa* was 512 μg/mL. The effect of EGCG on the growth of *P. aeruginosa* was determined by growth curves using sub-MICs, and no growth inhibition was found (Figure 2). We evaluated the impact of EGCG on biofilm formation and dissipation. Biofilm formation was significantly inhibited at time points of 24 h–48 h in a dose-dependent manner (Figure 3). The results indicated that EGCG could prevent biofilm formation without inhibiting the growth of *P. aeruginosa*.

### 3.3. EGCG Attenuated the Production of Pyocyanin

**Figure 4:**
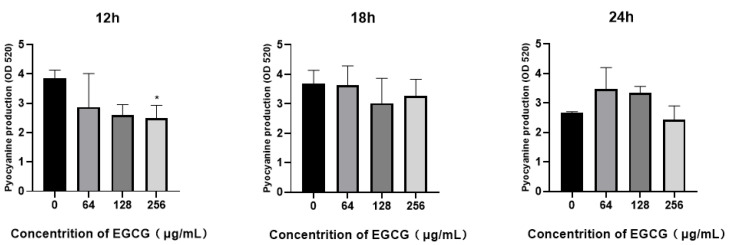
The inhibition of EGCG on *P. aeruginosa* pyocyanin production at 12 h, 18 h, and 24 h. (All data are representative of three independent experiments performed in triplicate and expressed as the mean  ±  SD values in each bar. * *p*  <  0.05.

Pyocyanin is a green exotoxin produced by *P. aeruginosa* and controlled by the intercellular process of bacterial communication known as quorum sensing. RhlR system orchestrates the generation of pyocyanin with the assist of PQS [50]. It plays an important role in human pulmonary infection; studies have shown that pyocyanin could increase the colonization and pathogenicity of *P. aeruginosa* during plant or animal infections [51,52,53]. We evaluated the inhibitory effect of EGCG on pyocyanin production. Moderate attenuation of *pyocyanin* production was noted at a concentration of 256 μg/mL after 12 h (Figure 4).

### 3.4. Protease Activity of P. aeruginosa Was Inhibited by EGCG

**Figure 5:**
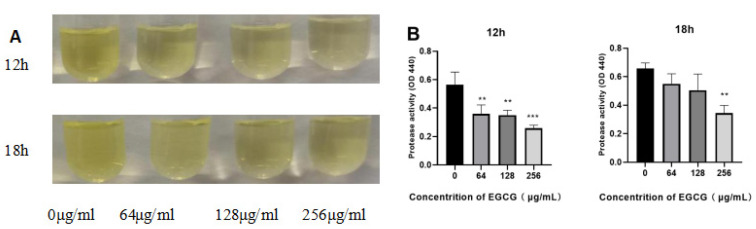
The inhibition of EGCG on protease activity in *P. aeruginosa*. (**A**) Images of the azocasein and virulent supernatant reaction product. (**B**) The quantitative estimated value of protease activity. (All data are representative of three independent experiments performed in triplicate and expressed as the mean  ±  SD values in each bar. ** *p*  <  0.01; *** *p*  <  0.001).

*P. aeruginosa* will secrete different virulence proteins such as proteases and elastases, propelling the pathogen to adhere, bring about damage to host tissue and blood vessel invasion [54,55]. As presented in Figure 5, protease activity was suppressed by EGCG with a dose-dependent effect. The suppression rate of protease activity was 54.74 and 46.22% for 12 h and 18 h at 256 μg/mL (*p* < 0.01). The inhibition rate was still maintained at 20.11% at the minimum concentration for 18 h. This implies a significant inhibiting effect of EGCG on protease activity of *P. aeruginosa*.

### 3.5. EGCG Inhibited Elastase Activity in P. aeruginosa

**Figure 6:**
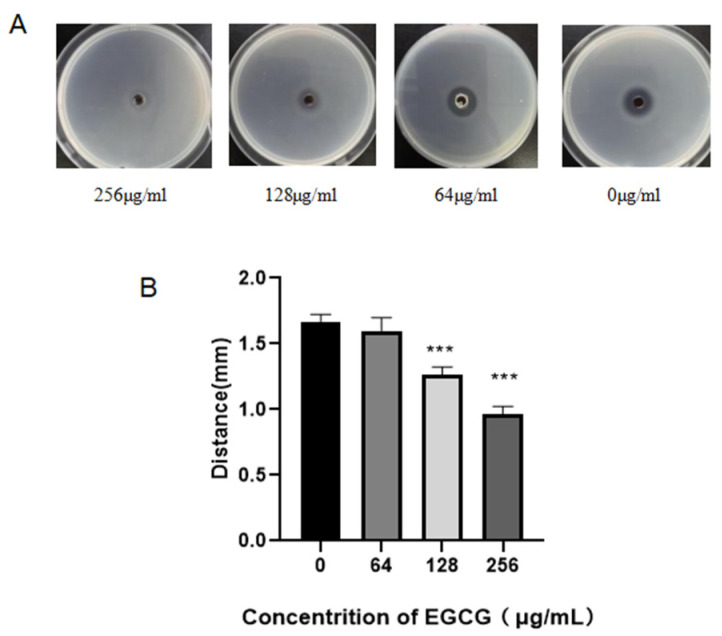
Concentration-dependent inhibition effects of EGCG on elastase activity in *P. aeruginosa*. (**A**). Using Skim milk agar plates to detect elastase activity. (**B**). The distance of transparent zones at 12 h and 18 h. (All data are representative of three independent experiments performed in triplicate and expressed as the mean  ±  SD values in each bar. *** *p*  <  0.001).

We assessed the elastase activity with the skim milk method, as shown by the reduction in the diameter of the transparent zones produced by *P. aeruginosa* on milk plates (Figure 6). The inhibition of EGCG on the elastase production presented with a dose-dependent effect. EGCG significantly inhibited elastase production compared with the control. This result suggested that EGCG could preclude the QS system of *P. aeruginosa*.

### 3.6. Swimming and Swarming Motility of P. aeruginosa Was Inhibited by EGCG

**Figure 7:**
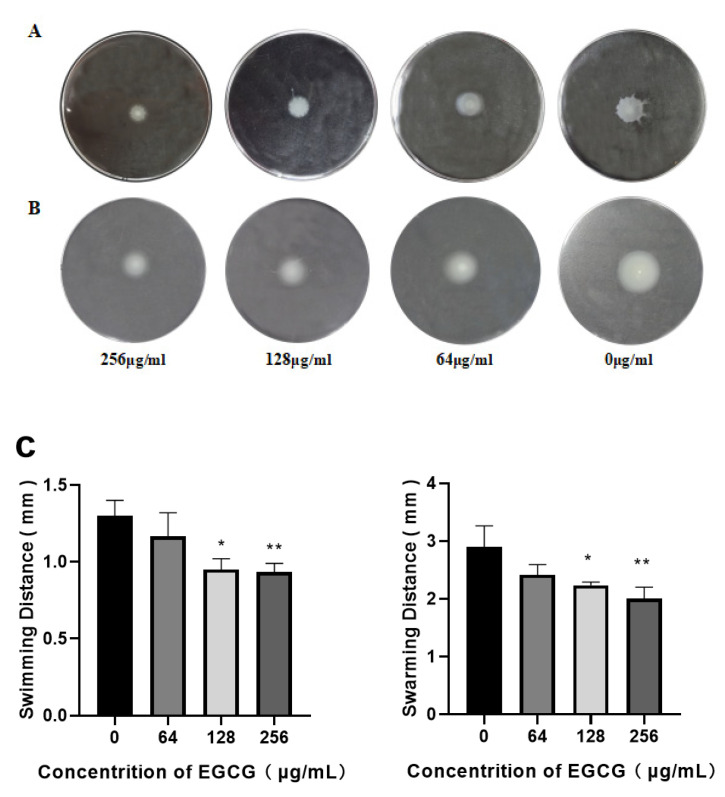
Motility abilities of *P. aeruginosa* on special agar plates. (**A**). Swimming, (**B**). Swarming, (**C**). The diameter of movement distance of *P. aeruginosa*. (All data are representative of three independent experiments performed in triplicate and expressed as the mean ± SD values in each bar. * *p* < 0.05; ** *p* < 0.01.

Bacterial motility also facilitates the formation of biofilms; swimming is derived from flagellum, which acts on the initial attachment of *P. aeruginosa*, the first step in biofilm formation [56]. Swarming motility impacts biofilm structure [57]. Both swimming and swarming were suppressed by EGCG in a dose-dependent manner, as exhibited in Figure 7.

### 3.7. EGCG Reduced the Production of Rhamnolipid

**Figure 8:**
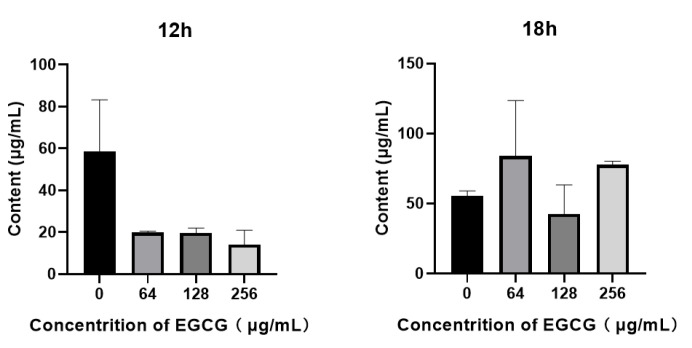
The inhibitory effect of EGCG on the production of rhamnolipid in *P. aeruginosa.* (All data are representative of three independent experiments performed in triplicate and expressed as the mean ± SD values in each bar.

Rhamnolipid is biosynthesized through rhamnolipid synthase rhlAB operon and rhlC, which is controlled by the rhlI/rhlR QS system [42]. The rhamnolipids facilitate surface motility of *P. aeruginosa* for biofilm formation and are also involved in the dispersal of mature biofilm [58]. Although Rhamnolipid production decreased at 12 h, there were no significant changes, and the rhamnolipid level did not exceed the control group except at a concentration of 128 μg/mL (Figure 8, 18 h).

### 3.8. EGCG Decreased the Production of C4-AHL

**Figure 9:**
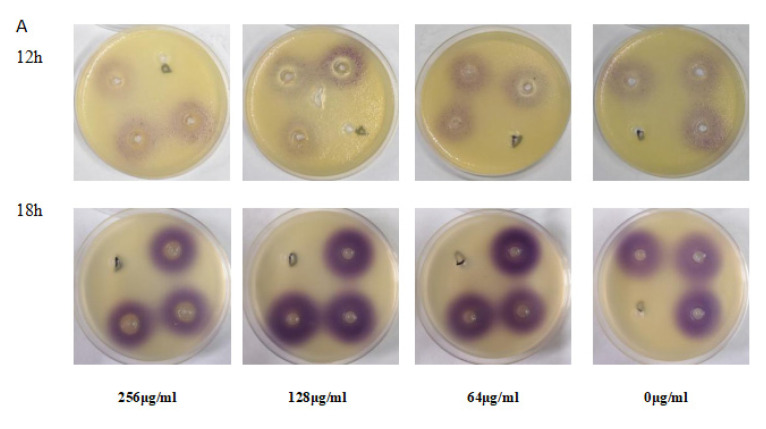

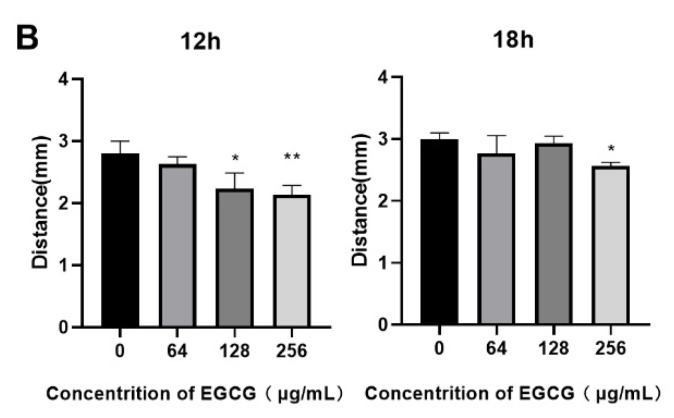
Effect of EGCG on the production of signaling molecules C4-AHL with the *C. violaceum* CV026. (**A**). Using LB agar with well diffusion assay to detect signaling molecules C4-AHL. (**B**). The diffusion distance of violacein at 12 h and 18 h. (All data are representative of three independent experiments performed in triplicate and expressed as the mean ± SD values in each bar. * *p* < 0.05; ** *p* < 0.01.

The effects of EGCG on the production of QS signaling C4-AHL were also examined via *C. violaceum*, by means of measuring the diameter of the purple zone evaluating the inhibition of QS signaling by EGCG. As showed in Figure 9A, the diameter of the purple zone in 18 h was larger than 12 h time points, which means more violacein was produced at 18 h. After 12 h of EGCG treatment (concentration 256 μg/mL), the purple area diameter was decreased, showing significant inhibition of effect on C4-AHL production. The activity was persistent after 18 h; however, a slight decrease was observed when tested on lower concentration (128 μg/mL) compared with control. These results demonstrated that the QS signal increased over time, and EGCG significantly inhibited the production of C4-AHL.

### 3.9. The Expression of QS Related Genes Inhibited by EGCG

**Figure 10:**
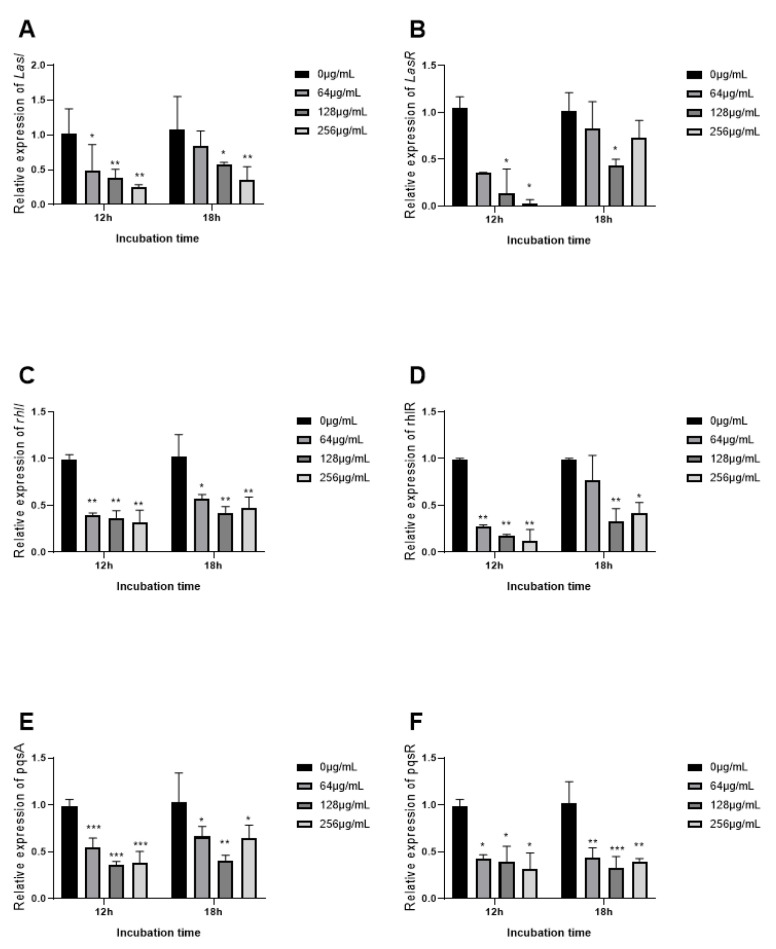
Effects of EGCG on the expression of QS genes compared with untreated control at 12 h and 18 h. ((**A**) LasI; (**B**) LasR; (**C**) rhlI; (**D**) rhlR; (**E**) pqsA; (**F**) pqsR). (All data are representative of three independent experiments performed in triplicate and expressed as the mean ± SD values in each bar. * *p* < 0.05; ** *p* <0.01; *** *p* < 0.001).

**Figure 11:**
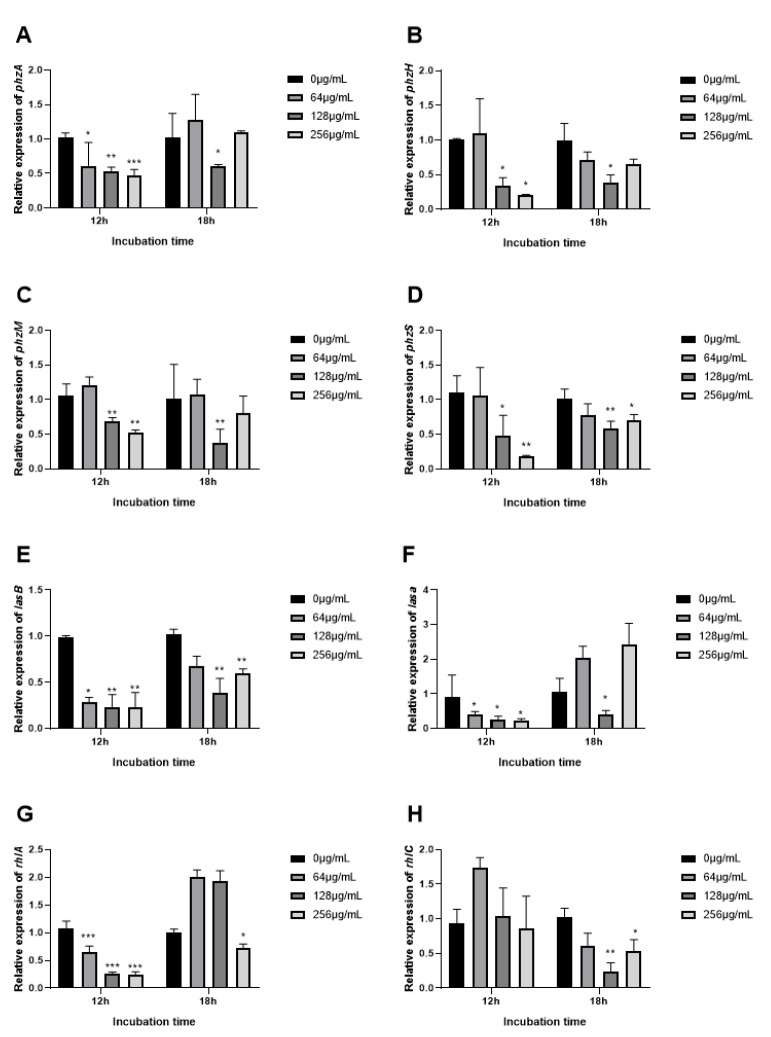
Effects of EGCG on virulence factors expression at 12 h and 18 h. ((**A**) phzA; (**B**) phzH; (**C**) phzM; (**D**) phzS; (**E**) LasB; (**F**) LasA; (**G**) rhlA; (**H**) rhlC). (All data are representative of three independent experiments performed in triplicate and expressed as the mean ± SD values in each bar. * *p* < 0.05; ** *p* < 0.01; *** *p* < 0.001).

To observe the inhibition mechanism of EGCG on *P. aeruginosa* virulence factors, we determined the expression of the QS gene measured by real-time quantitative PCR (RT-qPCR). QS regulatory genes (lasI, IasR, rhlI, rhlR, pqsA, and pqsR) and key QS-controlled genes (phzA, phzH, phzM, phzS, lasA, lasB, rhlA, and rhlC) in *P. aeruginosa* strains were investigated. The expression of a QS gene corresponded with the phenotypic experiments of virulence factors. Initially, a visible difference was observed in the QS signaling gene after 18 h as compared to 12 h of treatment. Expression with EGCG treatment rose at 18 h contrasted with 12 h. At 12 h QS -regulatory genes (lasI, lasR, rhlI, rhlR, pqsA, and pqsR) and key QS-controlled genes (phzA, phzH, phzM, phzS, lasA, lasB, rhlA, and rhlC) decreased in a dose-dependent manner, except for rhlI. EGCG significantly inhibited the expression of lasR, rhlR, pqsR, phzA, phzH, phzM, phzS, lasA, lasB, and rhlC at 128 μg/mL 18 h. EGCG suppressed transcriptional levels of lasI and LasR after 12 h, which indicates a decrease in the biosynthesis of protease and elastase as a manifestation of gene-product association with these genes were associated (Figure 10A,B), and the changes of relevant virulent genes LasA and LasB showed similar trends (Figure 11E,F). However, a significant reduction in the expression of LasR after 18 h of treatment was observed at 128 μg/mL with a marked reduction in LasA and LasB gene expression (Figure 10B and Figure 11E,F). Conclusively, EGCG significantly inhibited the expression of rhlR and rhlR-regulated virulence gene phzA, phzH, phzM, and phzS at 128 μg/mL 18 h (Figure 11A–D).

### 3.10. Supernatant Toxicity of P. aeruginosa in a C. elegans Model

**Figure 12:**
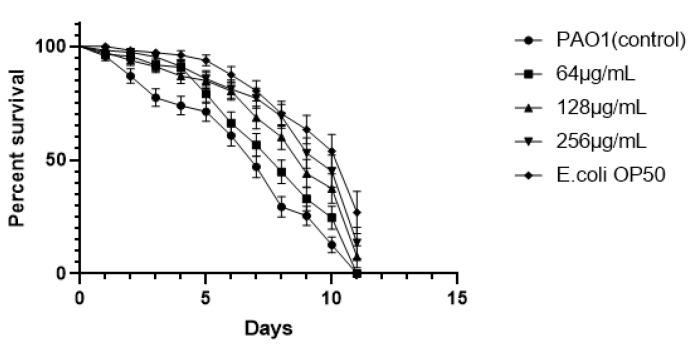
EGCG decreased the supernatant toxicity of *P. aeruginosa.* (All data are representative of three independent experiments performed in triplicate and expressed as the mean ± SD values in each bar.

*C*. *elegans* is a suitable model to study the pathogenicity of *P*. *aeruginosa* in-vivo due to its susceptibility to a variety of virulence phenotypes [59]. Herein, we investigated the supernatant toxicity of *P*. *aeruginosa* and the preventive effects of EGCG on the ability of the pathogen to kill *C*. *elegans*. As shown in Figure 12, after 11 days of incubation EGCG-treatment supernatant (256 μg/mL), the toxicity of *P. aeruginosa* was significantly reduced with an increase in survival rates of the infected *C. elegans* (N2) by 28.63% (*p* < 0.01). *C*. *elegans* was found dead in the *P*. *aeruginosa* supernatent control group. The survival rate of EGCG-treatment was maintained at 23.63% at 256 μg/mL, indicating an attenuation of the virulence supernatant of *P. aeruginosa* by EGCG.

### 3.11. Exogenous Supplementation of EGCG Prevents P. aeruginosa Killing of C. elegans

**Figure 13:**
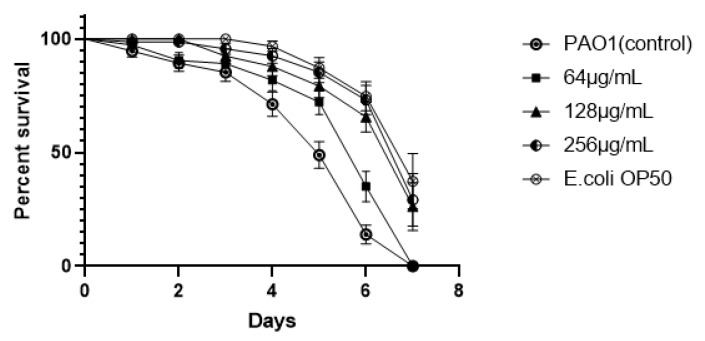
EGCG increased the survival rate of *C. elegans* infected with *P. aeruginosa.* (All data are representative of three independent experiments performed in triplicate and expressed as the mean ± SD values in each bar.

The suppressive effect of EGCG on *P. aeruginosa* infection was further assessed in-vivo. As showed in Figure 13, EGCG treatment showed a significant increase in the percent survival of *C. elegans*. When the mortality of *C. elegans* reached 50% (day 5), the survival rates of EGCG treatments (256 μg/mL) were 85.28% as compared to the control (48.93%) (*p* < 0.01). This confers an increase in survival rates of infected *C. elegans* by 36.35 when with exogenous EGCG treatment %. In addition, the survival rate of *C. elegans* fed *E*. *coli* (OP50) was similar to that of the EGCG-treatment group at 256 μg/mL and 128 μg/mL, ultimately the percentage survival of treatment groups of 256 μg/mL and 128 μg/mL to *C. elegans* was 26.27% and 29.23%, respectively. These results indicated that EGCG protected *C. elegans* against *P. aeruginosa* infection, in accord with its in vitro anti-virulence effects.

## 4. Discussion

**Figure 14:**
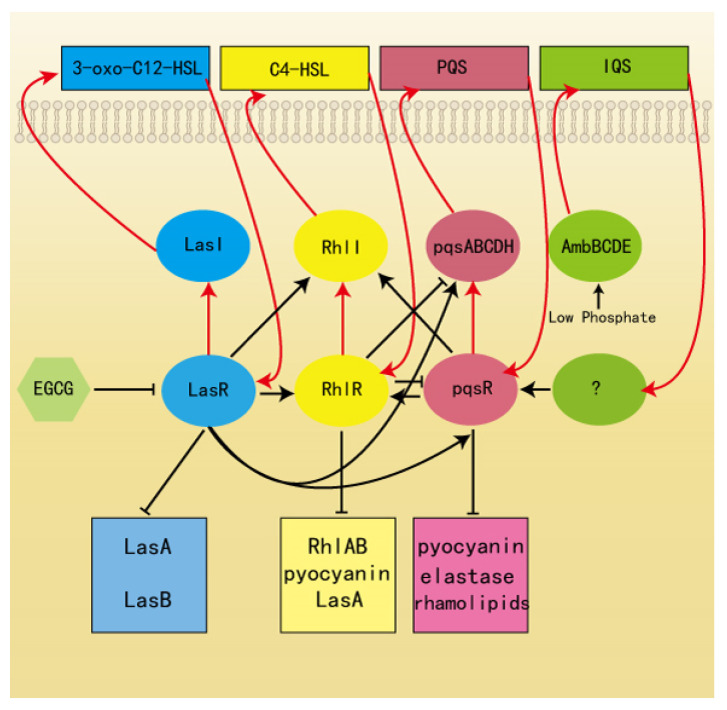
Quorum sensing circuits in *P. aeruginosa* and the action mechanism of EGCG.

Previous studies had determined the antimicrobial activity of EGCG and green tea extracts (GTE) against *P. aeruginosa* and *E. coli* isolated from skin wounds. EGCG and GTE have already shown promising activity as antibiotic adjuvants for infections that hard to treat for traditional antibiotic therapies [60]. Although the antibacterial activity of conventional antibiotics is generally higher than that of catechins, our interest in this group of natural products is due to the synergistic effect it has with a wide spectrum of antibiotics. Catechins have potential applications in clinical therapies due to the synergy, such as their ability to improve the sensitivity of antibiotics. A growing number of studies have revealed the antibacterial mechanism of catechins, inhibiting virulence, such as toxins and extracellular matrix molecules [61]. One study, for example, specifically reported that amyloid remodeling by EGCG changes the binding of QS molecules such as pyocyanin to the amyloid fibril, thus disrupting the QS system and inhibiting the production of extracellular matrix [62]. Structure-activity relationships (SARs) revealed that the activity of gallate moiety is necessary for α-synuclein and β-amyloid remodeling by EGCG [63], and the four major catechins (the levorotatory isomers of EGCG, EGC, ECG, and EC) indicate that EGCG and ECG are highly active in inhibiting *E*. *coli* biofilm than EC and EGC. This result indicates that the gallate moiety is essential for the antibiofilm activity of EGCG [64]. Herein, we report an effective application of EGCG as an anti-virulence agent. We were particularly aimed to evaluate the anti-QS activity of EGCG as an alternative approach for traditional antibiotic therapy. We confirmed that EGCG significantly suppresses the QS system without influencing *P. aeruginosa* growth. Further evidence of QS interference mechanisms was achieved via gene expression analysis, showing that EGCG efficaciously inhibited the transcriptional level of QS-regulated virulence factors. We proposed the primary target of EGCG is the receptor protein LasR (Figure 14). The relationship of four QS systems in *P. aeruginosa* is interactional; therefore, the other two QS systems RhlI and PQS were also inhibited. EGCG-treated *P. aeruginosa* expressed reduced virulence in *C. elegans* model. Our findings from gene expression experiments and with the animal model strongly suggest that EGCG holds convincing therapeutic potential for alleviation of *P. aeruginosa* infections.

QS inhibitors, in general, offer a promising alternative to antibacterial therapies, exerting lesser selective pressure for resistance compared with traditional antibiotics [65]. Therefore, targeting the regulation of virulence expression is a promising therapeutic option for the development of new therapies. Plant-based Natural products have been a great source of antimicrobial drugs. The anti-QS activity of EGCG was evaluated with *C. violaceum* (12472). The range of non-violacein halo gradually increased along with the increased concentration of EGCG. Hongping Yin et al. in 2015 proved the anti-QS activity of tea polyphenols (TP), which is a mixture of a compound consisting of four polyphenolic catechins. Their report displayed that TP had reduced violacein production by 82.56% in *C. violaceum* (at the concentration of 0.781 mg/mL) [44]. Here we found EGCG significantly reduced the production of violacein at concentrations as low as 4 μg/mL. EGCG significantly inhibited the development of biofilm (by 50.96% at 24 h) and pyocyanin (by 35.54% at 12 h). EGCG also demonstrated excellent protease inhibition activity by 54.74 and 46.22% at 12 h and 18 h, respectively. Vandeputte et al. reported that catechin had no significant effect on *P. aeruginosa* growth parameters, and catechin reduced biofilm formation by 30%, while EC had no effect. However, catechin and EC reduced the production of elastase by 30%. The inhibition of catechin for the expression of rhl system was stronger than the las system, which was consistent with phenotypic results in vitro. These results are not in agreement with our findings as we found that catechin acts by interfering with the perception of C4-HSL by RhlR; on the other hand, EGCG has presented a superior suppression effect to the las system than the rhl system at 12 h. The inhibition rates of EGCG for the expression of lasI, lasR, rhlI, and rhlR were also reported higher than catechin [66]. Therefore, EGCG is likely to be the most effective ingredient of catechin for QS inhibition.

Our results confirmed EGCG had no considerable effect on the growth of *P. aeruginosa*, and EGCG significantly suppressed the maturity and dispersion of biofilms. In addition, EGCG significantly inhibited the preliminary adhesion stage of biofilm (Figure 3). The biofilm development of *P. aeruginosa* is dynamic due to the changeable environment during this time by releasing different virulence factors promotes infection. QS also plays a significant role in biofilm development. The production of extracellular DNA (eDNA) is positively controlled by PQS; eDNA is contributed to the biofilm formation and the stability integrity of architecture [67]. Bacterial motility and the capacity of producing rhamnolipid are needed for the early stages of biofilm formation, which were coordinated by the rhl and PQS system circuits [57,68]. We detected the motility ability of *P. aeruginosa* with EGCG treatment. EGCG exhibited outstanding repression for the motility ability of *P. aeruginosa*. One of the known polyphenol compounds, Chlorogenic acid (CA), is abundant in fruits and vegetables, showing an inhibition rate of 33.3% [69]. By contrast, EGCG had a higher inhibition rate of 35.71%. However, EGCG had a better ability to inhibit swarming motility in comparison to swimming motility. This result was consistent with the significance of inhibition rate on the biofilm at different stages. Because swimming acts on the initial attachment of *P. aeruginosa* [56], and according to our biofilm inhibition experiment, the significant inhibition of initial adhesion was lower than the mature and dispersion period.

In our results, EGCG had no significant effect on the production of rhamnolipid, which had no obvious gradient changes. The reason may be that *P. aeruginosa* did not reduce enough rhamnolipid for detection due to short culture time and environment. To verify the results, we further determined the expression of rhlA and rhlC, which showed no significant inhibition on the expression of rhlA at 18 h, but the expression of rhlA significantly decreased at 12 h. The expression of rhlC presented a contrary tendency compared with rhlA. A previous study has shown that OligoG CF-5/20 only significantly reduced the production of rhamnolipid at 18 h, with no significant change seen at either 12 or 30 h [45], and our results are in line with these reports. These results indicated that the production of rhamnolipid was highly related to the growth period. A previous study reported that rhamnolipid synthesis in *P. aeruginosa* is regulated by the QS signaling system, and a reduction or increase in rhamnolipid and RhlR/RhlR can impact exopolysaccharide production, suggesting a plausible strategy by which QS signals may regulate biofilm matrix exopolysaccharides and swarming motility in bacterial communities in a coordinating manner [70]. Hence, the changes of biofilm, motility, and rhamnolipid showed some relevance.

Meanwhile, the pyocyanin and protease associated with QS are representative virulence of *P. aeruginosa*, which facilitates the early colonization of pathogens on host tissues and infection establishment. Thus, the pathogenicity of *P. aeruginosa* relies on the virulence factors [71]. As is well-known, *P. aeruginosa* possesses four clear hierarchical structure QS systems; these four systems have a cross control for the expression of the virulence factors. The activity of protease and elastase are collectively regulated by the las and rhl systems, and pyocyanin is regulated by the rhl and pqs systems [33,50]. Our results indicated that the inhibition of virulence factors by EGCG was time dependent. So, EGCG only inhibited the production of pyocyanin at 12 and 18 h. The activity of protease significantly decreased inhibition rate at 18 h by 46.22%. Chlorogenic acid displayed 37% and 30% inhibition for protease and rhamnolipid [69], and a previous study showed salicylic acid reduces the production of protease by 30% at 30 mM [72], later study shown protease had a significant reduction of 65% and 31%, respectively, with treatment with trans-cinnamaldehyde and salicylic acid, and CA and SA all significantly suppressed the rhamnolipid yield [73]. By contrast, EGCG has a more efficient inhibition on protease activity than pyocyanin/rhamnolipid. We speculated that EGCG has a great influence on 3-oxo-C12-AHL production than C4-AHL. Hence, we chiefly evaluated the C4-AHL production at 12 h and 18 h utilizing *C. violaceum* CV026 with a qualitative experiment, following treatment with EGCG (Figure 9A,B). The results presented distinct differences in C4-AHL production between 12 h and 18 h, which was highly related to the distance and degree of the purple halo, but the degree of the purple halo at 128 μg/mL was deeper compared with controls and the distance between 128 μg/mL and control was not changed.

To research the mechanism of the inhibiting discrepant of EGCG on different virulence factors, the expression of QS regulatory and virulence-associated genes at 12 h and 18 h were demonstrated. EGCG presented significant inhibition for relevant genes expression, including LasI, LasR, rhlI, rhlR, pqsA, pqsR, phzA, phzH, phzM, phzS, lasA, lasB, rhlA, and rhlC. The expression of these genes was significantly increased at 18 h compared to 12 h in a cell density-dependent manner. Our results are consistent with previous research [45,73], which selected four different time points from the logarithmic phase to the mid-late stationary phase of *P. aeruginosa*. Their results showed that the expression of QS genes achieved the largest level at the mid-late stationary phase. OligoG CF-5/20 had no significant effect on AHL expression at 30 h, and AHL expression appeared to decline [45]. Interestingly, the expression of autoinducers receptor lasR and rhlR significantly decreased at 18 h at the concentration of 128 μg/mL. Meanwhile, the expression of virulence factors is activated by the combination of LasR–3-oxo-C12-AHL and RhlR–C4-AHL complex [74]. These virulence factors significantly declined at 128 μg/mL. Our results indicated that the expression of virulence factors was strictly regulated by QS genes. EGCG could inhibit the expression of these genes and effectively control the production of virulence factors.

*C. elegans* has been universally utilized as an animal model system so as to investigate the anti-infective capacity of natural compounds against pathogens [75]. Pyocyanin triggers lethal paralysis in *C. elegans*, ultimately bring about the death of the nematodes. In addition, the motility of *P. aeruginosa* facilitates the colonization and diffusion of the pathogen in the *C. elegans* gut as well [46,76]. EGCG attenuated the supernatant toxicity increasing the survival of *C. elegans*. The production of virulence factors such as pyocyanin, protease, elastase aggravated the toxicity of *P. aeruginosa* supernatant to *C. elegans*. EGCG also increased the survival of *C. elegans* by 23.25%, 30.04%, and 36.35% in a concentration-dependent manner in vivo. Baicalin improved survival of *C. elegans* by approximately 20% at LT50 (24 h). The LT 50 of *P. aeruginosa* (ΔlasI-ΔrhlI) was longer than the wild-type [76]. These results suggested that EGCG significantly attenuated the virulence of PAO1 in vitro and in vivo.

## 5. Conclusions

In conclusion, EGCG significantly inhibited the biofilm development without growth inhibition and significantly reduced the production of virulence factors, which consistent with the expression of QS regulatory genes. Moreover, EGCG significantly increased the survival rate of *C. elegans* exposure to supernatant or directly infected with *P. aeruginosa* in vivo by attenuating its pathogenicity. Hence, EGCG is a promising QSI to alleviate the infections caused by *P. aeruginosa*.

## Figures and Tables

**Table 1:** List of primers used in qRT-PCR.

Target Name	Type	Primer Sequence
lasI	Fw	CGCACATCTGGGAACTCA
	Rev	CGGCACGGATCATCATCT
lasR	Fw	CTGTGGATGCTCAAGGACTAC
	Rev	AACTGGTCTTGCCGATGG
rhlI	Fw	GTAGCGGGTTTGCGGATG
	Rev	CGGCATCAGGTCTTCATCG
rhlR	Fw	GCCAGCGTCTTGTTCGG
	Rev	CGGTCTGCCTGAGCCATC
pqsA	Fw	GACCGGCTGTATTCGATTC
	Rev	GCTGAACCAGGGAAAGAAC
pqsR	Fw	CTGATCTGCCGGTAATTGG
	Rev	ATCGACGAGGAACTGAAGA
phzM	Fw	ACGGCTGTGGCGGTTTA
	Rev	CCGTGACCGTCGCATT
lasA	Fw	CTGTGGATGCTCAAGGACTAC
	Rev	AACTGGTCTTGCCGATGG
lasB	Fw	AACCGTGCGTTCTACCTGTT
	Rev	CGGTCCAGTAGTAGCGGTTG
phzA	Fw	AACGGTCAGCGGTACAGGGAAAC
	Rev	ACGAACAGGCTGTGCCGCTGTAAC
phzH	Fw	GCTCATCGACAATGCCGAACT
	Rev	GCGGATCTCGCCGAACATCAG
phzS	Fw	CCGAAGGCAAGTCGCTGGTGA
	Rev	GGTCCCAGTCGGCGAAGAACG
RhlA	Fw	TGGCCGAACATTTCAACGT
	Rev	GATTTCCACCTCGTCGTCCTT
RhlC	Fw	GCCATCCATCTCGACGGAC
	Rev	CGCAGGCTGTATTCGGTG
PvdQ	Fw	GCCGAGGAGATCGTCACC
	Rev	CAGGCGTAGAAGATGTCGGA

## Data Availability

The data presented in this study are available on request from the corresponding author.
